# Supplementary figures and images for: New insights into the criteria of functional heterozygosity of the Apis mellifera complementary sex determining gene–Discovery of a functional allele pair differing by a single amino acid
Source: PLoS One. 2022 Aug 9;17(8):e0271922. doi: 10.1371/journal.pone.0271922 (PMC9362917; doi:10.1371/journal.pone.0271922)

# Supplementary file S4

Uncropped images used in Figure 5

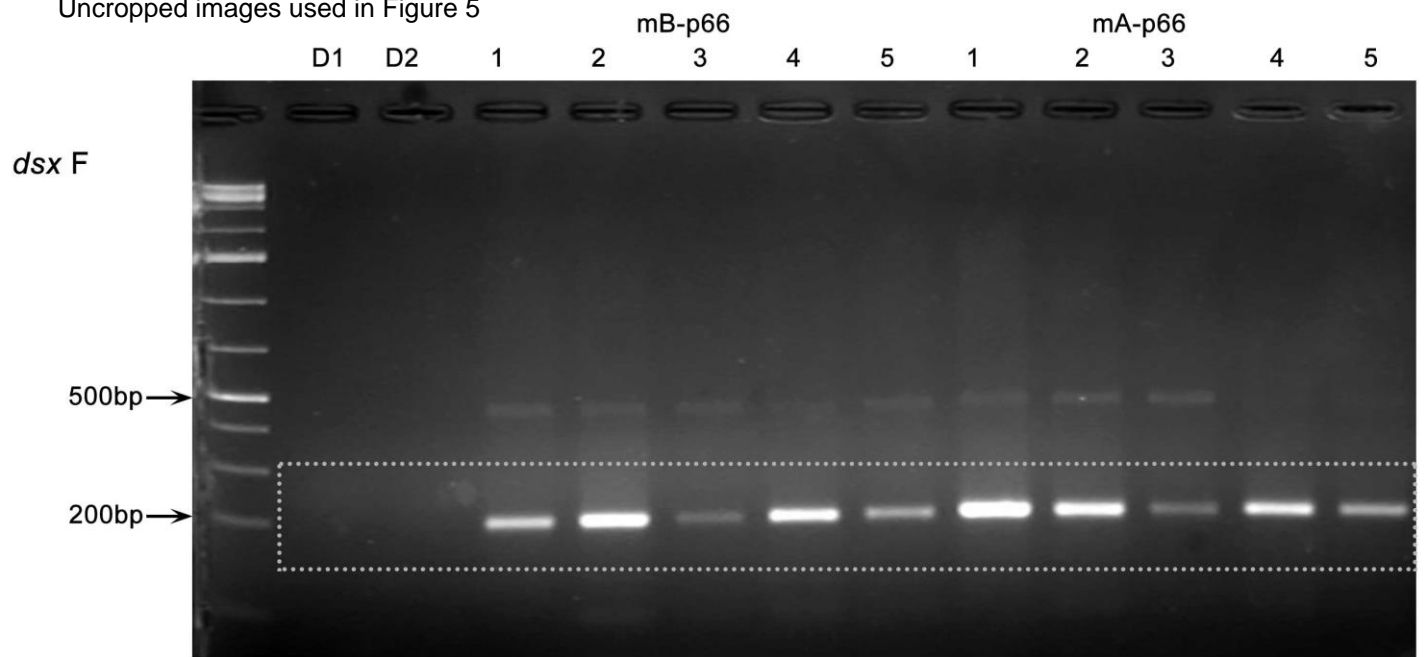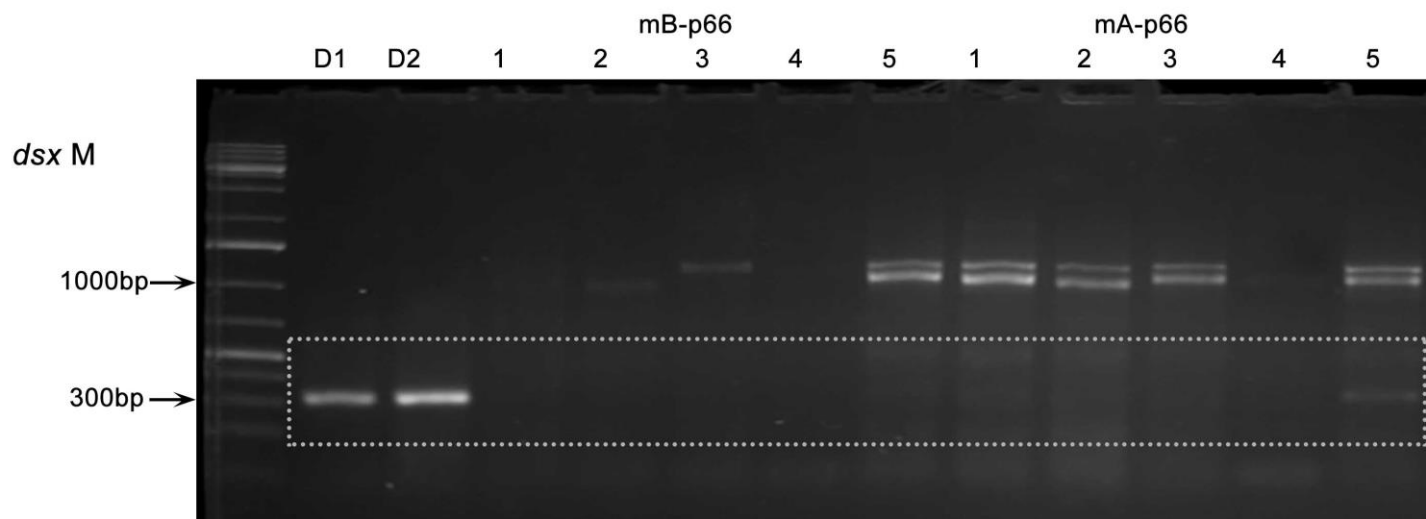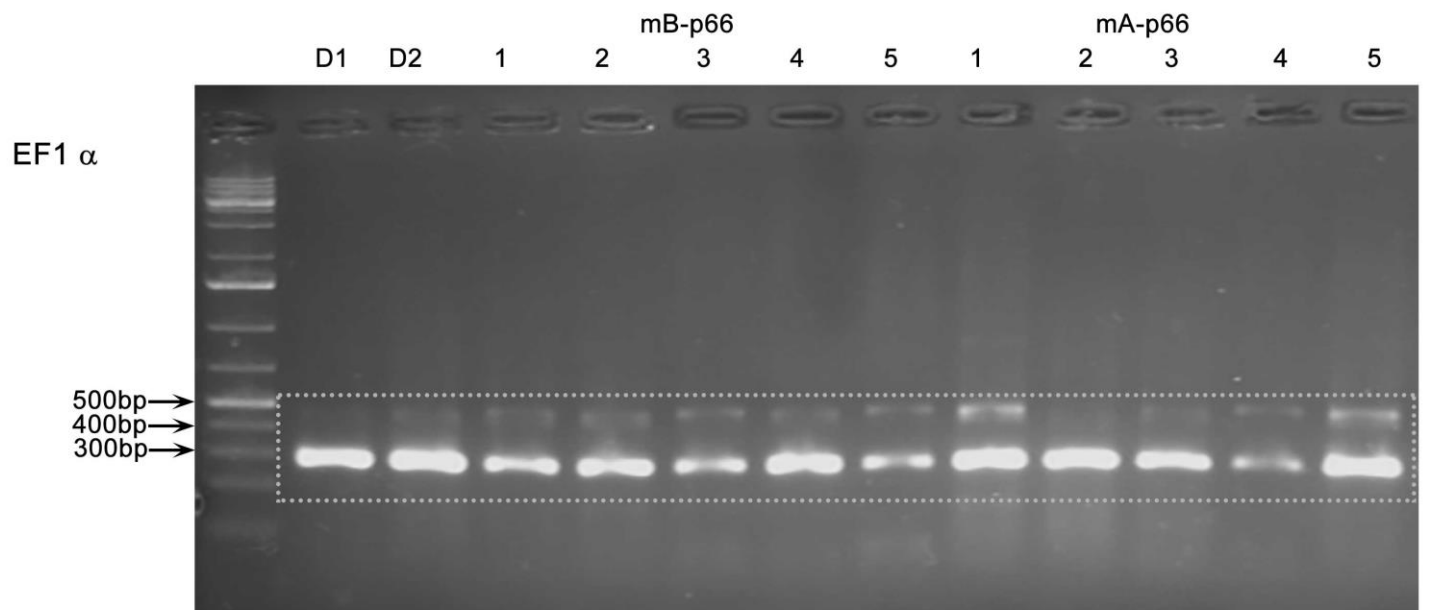

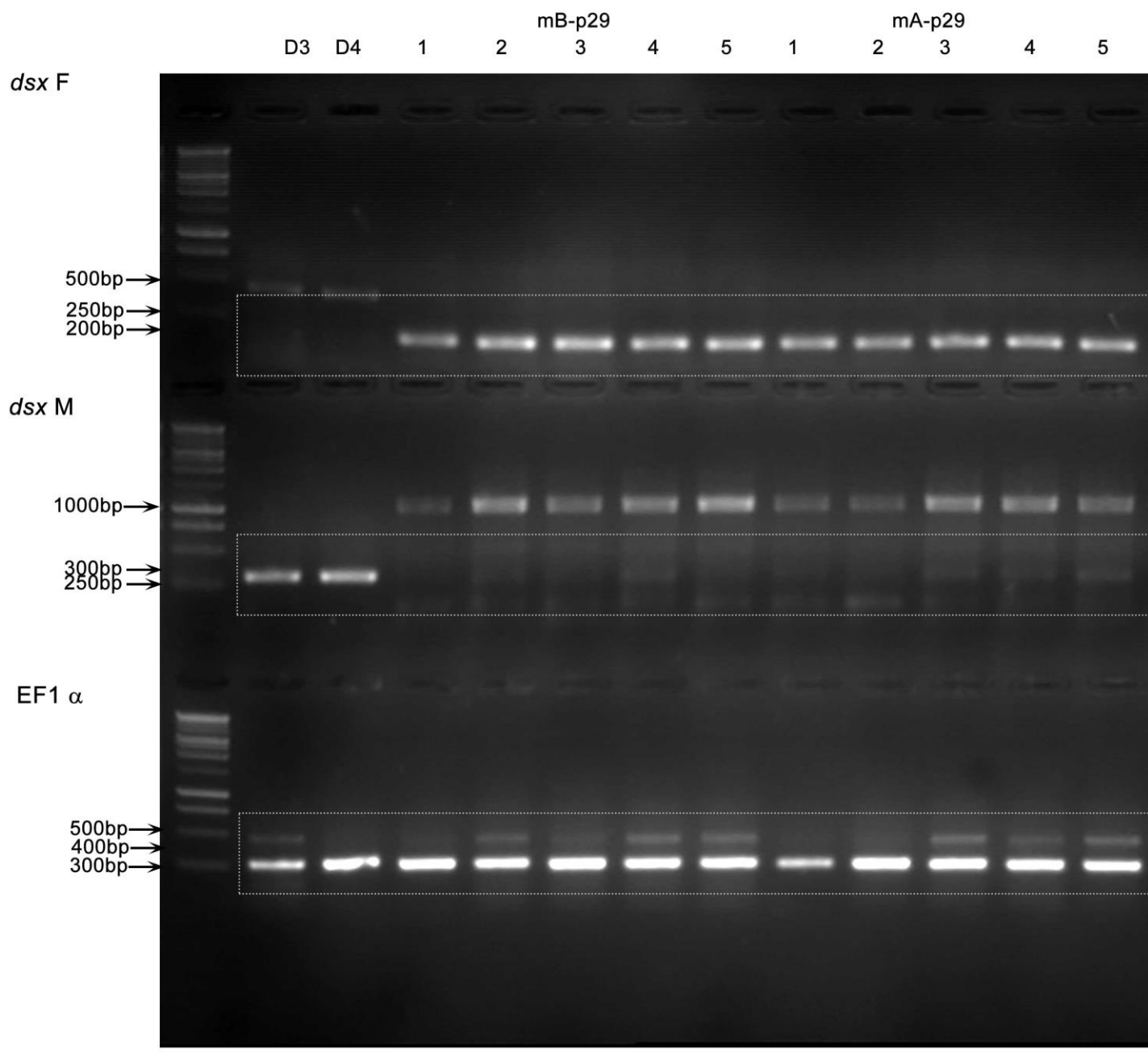

Supplement: S1 Raw images — (PDF) [file pone.0271922.s004.pdf]
