# Supplementary material for: New insights into the criteria of functional heterozygosity of the Apis mellifera complementary sex determining gene–Discovery of a functional allele pair differing by a single amino acid
Source: PLoS One. 2022 Aug 9;17(8):e0271922. doi: 10.1371/journal.pone.0271922 (PMC9362917; doi:10.1371/journal.pone.0271922)
Supplement: Attachment — Submitted filename: response.docx [file pone.0271922.s005.docx]

Dr. Christoph Englert	Wroclaw, 19.05.2022
Academic Editor
PLOS ONE

Dear Dr. Englert,

attached please find our response to the Reviewers’ comments regarding our paper: “Revisiting the criteria of functional heterozygosity of the *Apis mellifera* complementary sex determining gene” which we would like to publish in *PLOS ONE*. I hope you will find that we have adequately responded to the comments, made appropriate corrections and that our paper can be now accepted for publication.

Below please find our point-by-point response.

Kind regards – Malgorzata Cebrat

**Reviewer 1**

**1.** *Line 80-81: There is no definition for dPSD, just dPSD*

**Answer**: the definition of dPSD has been included in line 79.

**Reviewer 2**

**1.** *Line 47 - references should be reported. As far as I know, two publications list the number of csd alleles*

*Bilodeau, L., & Elsik, C. (2021). A scientific note defining allelic nomenclature standards for the highly diverse complementary sex-determiner (csd) locus in honey bees. Apidologie, 52(4), 749-754.
Bovo, S., Ribani, A., Utzeri, V. J., Taurisano, V., Schiavo, G., Bolner, M., & Fontanesi, L. (2021). Application of Next Generation Semiconductor-Based Sequencing for the Identification of Apis mellifera Complementary Sex Determiner (csd) Alleles from Honey DNA. Insects, 12(10), 868.*

*These works should be cited*

**Answer:** The above-mentioned papers were cited as references 6 and 7, respectively (lines 45 and 108)

**2.** *The aim of the study is not completely clear - a methodology to enabling the analysis of a large number of the csd genotypes of worker bees in order to identify those that are underrepresented or absent in bee colonies, under the assumption that these genotypes induce, at least to some extent, lethality - was used but the purpose is not well defined as the results were not as the authors would have assumed*

*It seems that the study is divided in two part: one that would like to establish if there would be any underepresented genotypes and the other that analysed the minimum sequence differences between the pairs to define lethality or not.*

**Answer:** Indeed, we agree that the aim was not completely clear and the results were presented in a confusing way. The aim of this study was to acquire information about a large number of *csd* genotypes of diploid individuals and to identify, whenever possible, genotypes that are underrepresented or absent in bee colonies in order to verify/refine the criteria of functional heterozygosity of *csd* alleles. We have now clarified this in the Introduction (lines 89-94) and Results section (lines 115-118). Also, the Results section had been rearranged to remove the misleading impression of the aforementioned division and to emphasize the importance of identifying *csd* genotypes in analyzed colonies (subsection *Identification and characterisation of csd genotypes*).

**3.** *A figure reporting the schematic representation of the methodology applied would help to follow the different steps and concepts*

**Answer:** The figure showing the schematic representation of the methodology is included in the revised version of the manuscript as Figure 2.

**4.** *line 118: a figure reporting an example of the pattern observed with the T-RFLP (terminal restriction length polymorphism)-based method is needed to clarify the methodology and the way in which this approach was able to identify paternal alleles*

**Answer:** The requested figure is included in the revised version of the manuscript as Figure 3A.

**5.** *Results were not really conclusive - this might be due to the fact that only five colonies were sampled - in addition it is not known when pupae (in which part of the year were sampled) - according to the ovideposition of the queen there would be potentially some biases in the distribution of the paternal alleles over the ovideposition period/years of the queen - this bias has not been taken into account to discuss the first part of the results - the biased genotypes*

**Answer:** The pupae were collected in early spring (April) and they were offspring of 1-year-old naturally inseminated queens. This information is provided in the Methods section (lines 413-414). We agree that there are biases in the distribution of the paternal alleles over the ovideposition period/years of the queen (as it has been hypothesized – e.g. due to improper mixing of spermatozoa in the spermatheca, Brodschneider et al, 2012), however, we would like to draw the Reviewer's attention to the fact that we have not attempted to compare the frequency of occurrence of paternal alleles in the analysed colony but the potential biases in the frequency of pairing of a given paternal allele with the two maternal alleles present in the colony. Figuratively speaking, we did not pay attention to differences in the number of worker bees identified in the patrilines (differences in the heights of the bars in Fig 3C) but we have analyzed each patriline separately in order to establish whether there was a difference in pairing with the maternal alleles (comparing blue vs orange parts of each bar). This fact has been emphasized in lines 121-124 of the revised version of the manuscript.

**6.** *Some concluding remarks are missed - what is the final message derived by t he obtained results ? what are the limits of the approach? this should be reported in the discussion and conclusion as well as in the abstract*

**Answer:** The final message of the manuscript is the identification of a fully functional *csd* pair differing by one amino acid residue and the resulting conclusion that criteria for functional heterozygosity of the *csd* gene are highly dependent on the sequence context and position of the change and the generation of a new *csd* specificity may occur during a single mutation step with no need for evolutionary intermediates accumulating further mutations. This message has been now included in the Abstract (lines 26-30) and the final part of the Discussion (lines 379-385). The limitations of the method implemented in our research – low efficiency in identifying potential biases in the frequencies with which a given paternal allele pairs with the maternal alleles (approx. 50% of the patrilines had to be excluded from the analysis due to the insufficient number of individuals needed to draw statistically meaningful results) has been now presented in the Results and Discussion sections (lines 207-211 and 301-313, respectively).

**7.** *All elements related to the simulation study is missed - it is described partially in results - however, it is not clear how csd sequence data were retrieved, when, and how have been filtered - Actually a much larger number of alleles than those used in the simulation. All different steps of the simulation part should be described here. It is not very clear how virtual spermateca have been constructed and the potential biases due to unequal sperm representation have been considered in the simulation*

**Answer:** Details of the simulation have been now included in subsection “Simulation of the number of the restriction patterns of csd alleles present in spermathecas” of the “Methods” section (lines 388-410). a) Yes, it is true that the databases contain much more records on *Apis mellifera csd* allele sequences – our most recent search (May 2022) retrieved 667 records. Regrettably, because most of the research is focusing on the hypervariable region alone, only 317 records contained the full sequence needed for our analysis (depicted in Figure 1 of the manuscript). After elimination of duplicates (317->214) and the records containing obvious errors in sequencing (out of frame indels in exon sequences) we have now been able to identify 151 distinct *csd* alleles on the level of nucleotide sequence. Those 151 alleles represented 79 distinct restriction patterns. We have now redone our simulation using the updated set of sequences. b) Because the aim of this simulation was to estimate the difference between the absolute number of the distinct alleles present and the number of distinct restriction patterns they represent, in our opinion it is not necessary to consider biases in sperm representation in the spermatheca. In other words – assuming that the queen mated with a given number of drones we wanted to check how many distinct alleles and restriction patterns are present in her spermatheca but not how they will be distributed in the worker bees.

**8.** *How multiple testing was considered?*

**Answer:** Testing for statistically significant differences between the expected frequencies of the presence of maternal alleles (0.5/0.5) and the observed frequencies within a given patriline is a problem of the simultaneous testing of more than one hypothesis i.e. testing of several patrilines within a colony . Therefore, Pearson chi-squared tests with Bonferroni correction for multiple (i.e. the number of tests performed for a given colony) testing (R statistical package) was used to adjust p-values to avoid the problem of higher probability of false rejecting the general null hypothesis, i.e. there is no significant differences in all considered cases.

This information has now been included in the manuscript (lines 473-479)

**9.** *It is not clear if the authors defined the minimum number of sequences to be obtained from workers to infer with a sound statistical approach if there would be any biases in the paternal allele distribution - this part is quite naïve*

**Answer:** We have assumed that the queens mated with approx. 15 drones and that the minimal size of the patriline group needed to identify potential biases in pairing with the maternal alleles within a given patriline as 16, thus we have estimated the number of individuals needed for the analysis as 240. This information has been now included in the Methods section (lines 414-417). We have not been able to predict the extent of the bias in sperm distribution and we admit in the revised version of the manuscript that this assumed number was too small, since many patrilines groups had to be excluded from the analysis due to the insufficient number of individuals. In the revised version of the manuscript we have also emphasized the usefulness of our approach in identifying genotypes in the analysed colonies.
