# Supplementary material for: New insights into the criteria of functional heterozygosity of the Apis mellifera complementary sex determining gene–Discovery of a functional allele pair differing by a single amino acid
Source: PLoS One. 2022 Aug 9;17(8):e0271922. doi: 10.1371/journal.pone.0271922 (PMC9362917; doi:10.1371/journal.pone.0271922)
Supplement: Attachment — Submitted filename: response.docx [file pone.0271922.s006.docx]

Dr. Christoph Englert	Wroclaw, 6.07.2022
Academic Editor
PLOS ONE

Dear Dr. Englert,

attached please find our response to the Reviewer comments regarding our paper: “New insights into the criteria of functional heterozygosity of the *Apis mellifera* complementary sex determining gene – discovery of a functional allele pair differing by a single amino acid” (former title “Revisiting the criteria of functional heterozygosity of the *Apis mellifera* complementary sex determining gene”) which we would like to publish in *PLOS ONE*. I hope you will find that we have adequately responded to the comments, made appropriate corrections and that our paper can be now accepted for publication.

Below please find our point-by-point response.

Kind regards – Malgorzata Cebrat

**1.** *“The title: based on the results obtained - jus one sequence that differ by one amino acid is a preliminary suggestion and and a confirmation of what is stated”*

**Answer:** The title has been changed to precisely describe our discovery and their possible impact on the matter of functional heterozygosity of *csd* gene.

**2)** *“The abstract is largely written including introductions to the context -no M&M info and just a few lines of results are reported - it should be better balanced - please take also into account that too bold conclusions should be better balanced.”*

**Answer:** The abstract has been rewritten to accommodate Reviewer’s suggestions – the introduction part has been shortened to a minimum needed to understand the context of our work, the M&M information has been incorporated into the description of the results and the conclusions are, in our opinion, better balanced. Please also note that the final sentence of the Discussion part has been added to better balance our conclusions.

**3)** “*In Table 2: They are not clear the the numbers corresponding to the "frequency of maternal alleles*"

**Answer:** An additional line has been added to the Table 2 legend in order to clarify the issue. We have also added the information which maternal alleles (A to G) were present in a given colony and how many worker bees carried it.

**4)** *“Not clear the nomenclature reported at line 232”*

**Answer:** The description of the genotypes have been changed (lines 237-239)
